# A Novel Voltage Gradient Configuration Strategy for 200 t/d Oxygen-Fuel Combustion Coupled Electric Boosting Glass Melting Systems

**DOI:** 10.3390/ma19040651

**Published:** 2026-02-08

**Authors:** Xurong Teng, Dinghao Yang, Ouyuan Zhang, Lin Yuan, Fangfang Zhao, Changyuan Tao, Renlong Liu

**Affiliations:** 1 College of Chemistry and Chemical Engineering, Chongqing University, Chongqing 400044, China; txr20250701@163.com (X.T.); 202418021059@stu.cqu.edu.cn (D.Y.);; 2 State Key Laboratory of Coal Mine Disaster Dynamics and Control, Chongqing University, Chongqing 400044, China

**Keywords:** fiberglass, furnace, electric boosting, combustion coupled electric boosting, voltage, computational fluid dynamics

## Abstract

The production of high-performance glass fibers relies critically on achieving a homogeneous melt with a specific thermal history, which is directly determined by the precise control and optimization of the melting equipment. To enhance the melting efficiency and material quality, this study investigates the optimization of the electric assistance system in a 200 t/d oxygen-enriched glass fiber melting furnace. By integrating CFD (Computational Fluid Dynamics) simulation techniques, a furnace model encompassing both the combustion zone and molten glass phase is developed. The study focuses on the impact of an oxy-fuel combustion + electric assistance system on the glass melting process. The influence of different input voltages on the furnace is analyzed through temperature, velocity, and flow fields. Glass melting efficiency and quality are evaluated using residence time, melting factor, and homogenization factor, considering both the residence time of molten glass and quality factors. The results indicate that a voltage scheme with the highest input voltage at the furnace inlet, combined with a relatively high voltage at the furnace outlet, is optimal, leading to the superior glass melting quality and the longest furnace service lifespan.

## 1. Introduction

The consistent manufacturing of high-quality glass fibers depends critically on the precise regulation of the melting equipment to ensure a homogeneous melt. In this context, the electric assistance melting process in fiberglass production plays a critical role. It involves inserting electrodes into the molten glass, where the Joule heating directly heats the glass melt. This process leverages the thermal conductivity of the glass melt to efficiently aid in the melting process [1,2,3,4]. In its molten state at high temperatures, glass exhibits electrical conductivity, allowing the Joule heat generated by the electric current to facilitate the efficient melting of the glass melt [5,6,7]. As an auxiliary method, the electric assistance melting process enhances the traditional combustion-based heating of glass furnaces by offering several advantages, including high thermal efficiency, good thermal stability, and uniformity. These benefits contribute to improved glass quality, increased melting capacity, and reduced energy consumption [8,9,10]. Furthermore, the use of electric energy, a clean energy source, significantly reduces pollutant emissions, playing a crucial role in achieving carbon peak and carbon neutrality goals.

A large body of research has been conducted by researchers on electric boosting melting. Hailong Li et al. [11] used the MHD module in ANSYS FLUENT 14.0 software to simulate and analyze two types of all-electric melting furnaces with capacities of 15t/d and 36t/d. They analyzed the distribution of electrical power density, temperature distribution, and velocity fields inside the two furnace models. It was found that the electrical power density and temperature first increase and then decrease in the horizontal direction from the furnace center to the sidewalls. A similar trend was observed vertically, from top to bottom. The temperature distribution leads to internal and external circulation in the all-electric melting furnace. McLaren, C et al. [12] studied the effect of external electric fields on glass viscosity. The results showed that the application of a DC electric field significantly lowered the softening temperature of glass, making electric field-induced softening (EFIS) significant. Luyao Li et al. [13,14] established an integrated glass furnace model that combines the combustion chamber and glass bath, studying two methods to increase glass production: increasing fuel supply and introducing electric boosting melting. The results indicated that, while ensuring glass quality and furnace lifespan, increasing fuel supply is a reasonable and effective method, which can increase glass furnace production to below 650 t/d and further raise it to around 700 t/d. Liu, ZY et al. [15] used the finite element method to calculate the dominant heat transfer and obtain the temperature distribution. They also applied the Smoothed Particle Hydrodynamics (SPH) method to model the melt flow and free surface deformation. The study examined the impact of heating conditions on the melting process. They proposed a furnace design with bottom heaters and optimized heating strategies to improve the melting process by suppressing incomplete melting phenomena. Dzyuzer, VY et al. [16] performed a numerical simulation of external heat exchange in gas-electric glass furnaces. The results showed that the added electric heating intensity of glass blocks influences the normal operation of the furnace. Kyeongjun Seo et al. [17] suggested that electric boosting melting improves thermal efficiency, reduces direct pollutant emissions, and extends the lifespan of the upper structure of the furnace. However, considering the volatility of electricity prices, high levels of electricity usage are not always economically feasible. They established a physically based model to describe the dynamic behavior of a prototype electric glass furnace. A dynamic optimization strategy was proposed to achieve an optimal balance between natural gas and electricity consumption under fluctuating electricity prices. Case studies analyzing the impact of different energy prices and emission regulations were conducted. Joaquin Garrido-Zafra et al. [18] used a mixed binary quadratic programming problem to build an optimal power load scheduling strategy that avoids the degradation of power quality, while also considering power quality and thermal behavior guidelines. Jianjun Han et al. [19] introduced an electric boosting melting system into a float glass furnace, studying the effect of different electrode positions on the glass melting process. The results showed that when the electrodes are installed below the intermittent layer, an accelerated intermittent melting process and stable convective flow could be achieved, further improving the glass quality and melting efficiency of the furnace. Georg Daurer et al. [20] compared different clarification technologies (electric boosting melting and bubbling) in industrial furnaces, including their effects on temperature, melt flow patterns, glass quality, heat flux, and process efficiency. The results indicated that both methods significantly affect the total energy conversion inside the furnace. Due to the induced surface temperature drop, bubbling achieved almost the same total energy input to the glass bath as electric boosting melting.

However, a review of the literature reveals that while existing studies cover the physical fields of all-electric furnaces, capacity enhancement in hybrid furnaces, electrode layout, and cost-based power scheduling, a significant research gap remains in the specific domain of “oxy-fuel glass fiber furnaces coupled with electric boosting.” Current coupled models often treat electric boosting as a lumped heat source or focus solely on optimizing total power and electrode placement, thereby overlooking the precise regulatory role of “input voltage gradient configuration” (i.e., the voltage allocation strategy across different regions).

In view of this, this paper establishes a complete Computational Fluid Dynamics (CFD) model using ANSYS Fluent based on a 200 t/d oxy-fuel glass fiber furnace with electric boosting. Unlike traditional studies that focus on electrode position or total power, this work targets the voltage gradient as a critical variable, designing and comparing six different voltage input schemes. By analyzing the temperature field, flow field, and melting/homogenization indices, this study aims to reveal the underlying mechanisms of how voltage distribution impacts melting quality, providing a theoretical basis for developing scientific electrode operation strategies.

## 2. Numerical Simulation

### 2.1. Glass Furnace Operating Conditions

**Figure 1:**
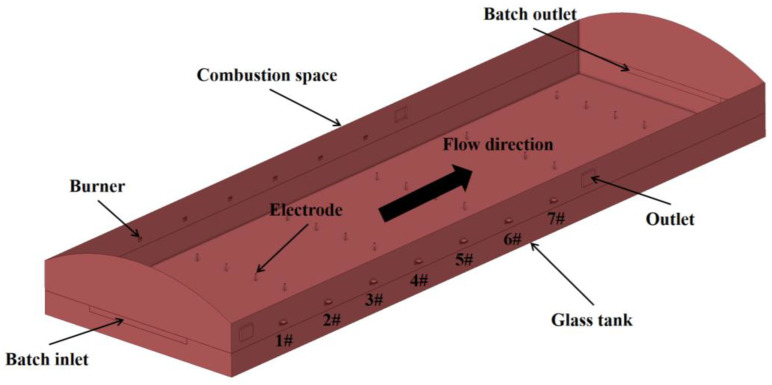
Three-dimensional schematic diagram of glass fiber furnace [21].

**Figure 2:**
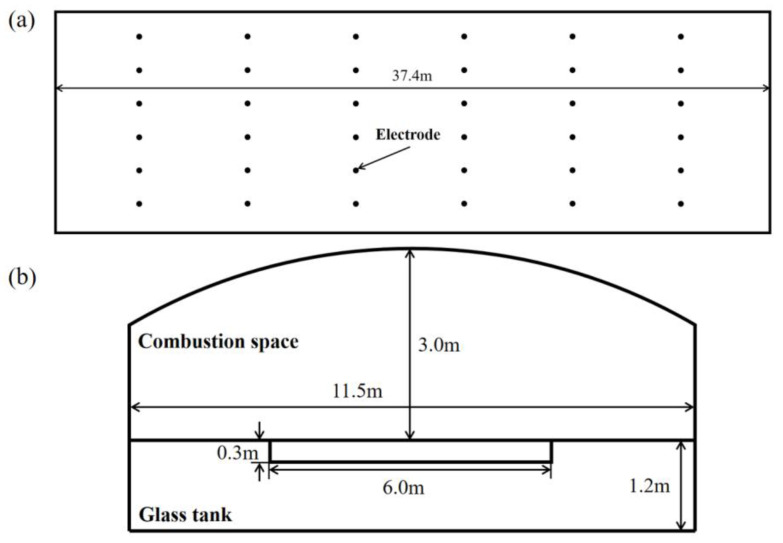
Size diagram of glass furnace. (**a**) Horizontal plane. (**b**) Side section [21].

Based on the production line of Chongqing Sanlei Glass Fiber Co., Ltd., (Chongqing, China), this study establishes a numerical model for a 200 t/d glass fiber furnace using ANSYS Fluent 2022R1 software. The furnace uses natural gas as fuel and oxygen as the oxidizing agent, with a natural gas consumption rate of 0.636 m^3^/s (at standard conditions: 273.15 K and pressure of 101.325 kPa). The furnace is equipped with seven pairs of burners, arranged in seven groups along the flow direction of the molten glass. Each group consists of a pair of symmetrically positioned burners, as illustrated in Figure 1. The fuel distribution for each burner is shown in Table 1. Figure 2 displays the specific dimensions of the model. In the model, the boundary conditions of the burner in the combustion space are set as a velocity inlet; in the glass pool furnace model, the material input boundary condition is set as a mass flow inlet, and the liquid flow channel outlet boundary condition is set as a pressure outlet. The ambient temperature is set at 30 °C, and the refractory material on the surface of the furnace has natural convection heat transfer boundary conditions with the environment. Electrodes are arranged with bottom insertion at the furnace’s bottom, with a total of six rows of electrodes, and each row consists of six electrode rods. Two rows of electrodes form a group, and the electrode rods heat the glass melt inside the furnace by applying constant power. The generated Joule heat, together with the heat produced by combustion, jointly affects the glass melt, generating different temperature fields at different locations within the melt, thus promoting the homogenization of the glass melt. This study investigates the impact of electrode voltage on the glass melting process by establishing a model for electric boosting melting with different electrode input voltages. The research scheme is shown in Table 2. Only the input voltage is varied, with all other conditions held constant, and thus the model and grid of each scheme remain unchanged.

The combustion gases are a mixture, and properties such as viscosity, thermal conductivity, and specific heat are defined by calculating the mass fraction averages of the pure substances. The density is calculated using the ideal gas law.

The study uses a typical Na-Ca-Si glass, and the thermal physical properties of the molten glass are shown in Table 3.

### 2.2. Numerical Model

In the glass melting furnace, the complex physical and chemical processes involved in the combustion space and glass pool furnace follow the laws of mass conservation, momentum conservation, and energy conservation. The control equations that need to be solved in the established numerical model of the glass fiber melting furnace are as follows:

Mass Conservation Equation:

(1) $$\frac{\partial \rho }{\partial t}+\nabla \cdot \left(\rho \vec{V}\right)=0$$

where ρ is the fluid density (kg/m^3^), *t* is the time (s), and $\vec{V}$ is the fluid velocity vector (m/s).

Conservation of momentum equation:

(2) $$\frac{\partial \left(\rho \vec{V}\right)}{\partial t}+\nabla \cdot \left(\rho \vec{V}\vec{V}\right)=-\nabla P+\nabla \cdot \left(\mu \nabla \vec{V}\right)+\rho \vec{g}$$

where *P* is the static pressure (Pa), μ is the dynamic viscosity of the fluid (Pa·s), and $\vec{g}$ is the gravitational acceleration vector (m^3^/s). The external body force term is represented solely by gravity in this study.

Energy Conservation Equation:

(3) $$\frac{\partial \left(\rho C_{p}T\right)}{\partial t}+\nabla \cdot \left(\rho \vec{V}C_{p}T\right)=\nabla \cdot \left(k_{eff}\nabla T\right)+S_{rad}+S_{Joule}$$

where $C_{p}$ is the specific heat capacity (J/(kg·K)), *T* is the temperature (K), $k_{eff}$ is the effective thermal conductivity (W/(m·K)), $S_{rad}$ is the radiative heat source term (W/m^3^) representing the divergence of the radiative heat flux calculated by the DO model, and $S_{Joule}$ is the electrical heat source term (W/m^3^).

For the electric boosting melting process, the electric potential distribution is governed by the conservation of charge, as shown in Equation (4):

(4) ∇·(σ∇φ)=0

where φ denotes the electric potential, σ is the temperature-dependent electrical conductivity of the molten glass (Ω^−1^·m^−1^).

The generated volumetric Joule heat source ($S_{Joule}$), which is coupled into the energy equation, is calculated according to Equation (5):

(5) $$S_{Joule}=\sigma {\left|\nabla \varphi \right|}^{2}$$

where $S_{Joule}$ represents the heat generation rate per unit volume derived from the electric field intensity and the conductivity of the glass melt.

In this study, the ANSYS Fluent 2022R1 software was used to establish a melting furnace model that includes both the combustion space and the glass melt. Specifically, within the combustion chamber, the k-ε model, species transport, and radiation model (DO) were employed to study the flow and other behaviors in this space. In the glass pool furnace, the model was developed using laminar flow, the radiation model (DO), and electric boosting melting in the form of Joule heating. The coupling interface (the surface of the glass melt) between the combustion chamber and the glass pool furnace facilitates the exchange of data during the numerical simulation.

## 3. Results and Discussion

### 3.1. Mesh Independence Verification

**Figure 3:**
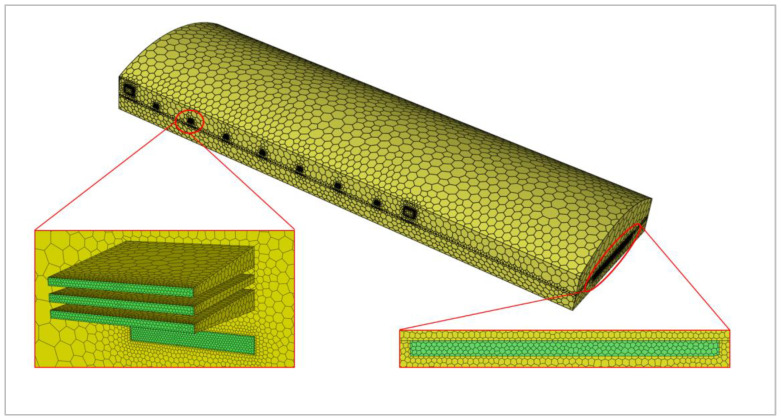
Schematic diagram of grid division of glass furnace.

Figure 3 shows the mesh generation of the furnace with a minimum mesh size of 5 mm. The computational domain includes the glass tank and combustion space. A polyhedral mesh was used with refinement at the inlets and outlets, resulting in a total mesh count of 1,358,950. In terms of mesh quality, the minimum orthogonal quality is 0.21, the average orthogonal quality is 0.94, and the average skewness is 0.0599, meeting the requirements for numerical accuracy.

Based on the mesh generation results, the computational domain consists of a total of 1,358,950 polyhedral cells. Local mesh refinement was applied to the inlets and outlets of both the combustion gas and the glass.

**Figure 4:**
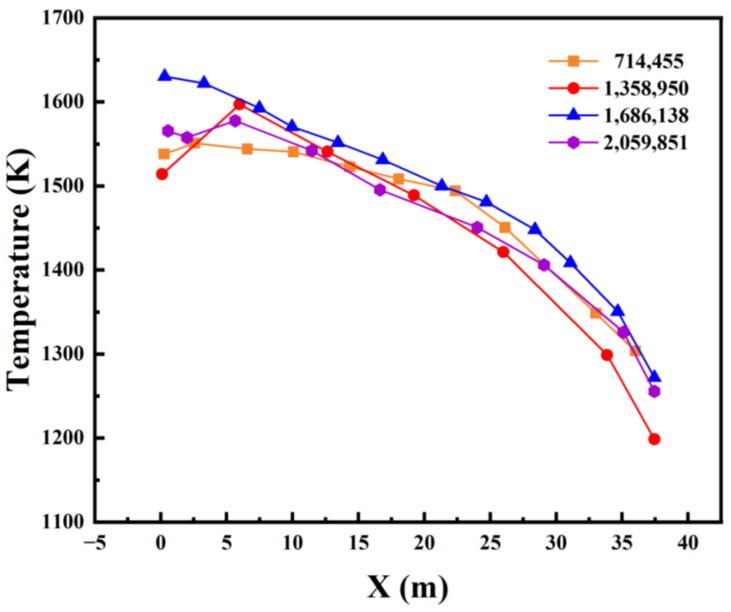
Bottom temperature of glass furnace.

To evaluate the influence of mesh density on the simulation results, a grid independence test was conducted using four different mesh systems with cell counts of 714,455, 1,358,950, 1,686,138, and 2,059,851. As shown in Figure 4, the calculation results tend to stabilize as the number of grid cells increases. The mesh with 1,358,950 cells effectively captures the temperature field characteristics in critical regions and shows good agreement with the higher-density meshes. Therefore, balancing computational accuracy and efficiency, the mesh system with 1,358,950 cells is considered appropriate for the simulation.

### 3.2. Joule Heat Density Distribution and Temperature Distribution

**Figure 5:**
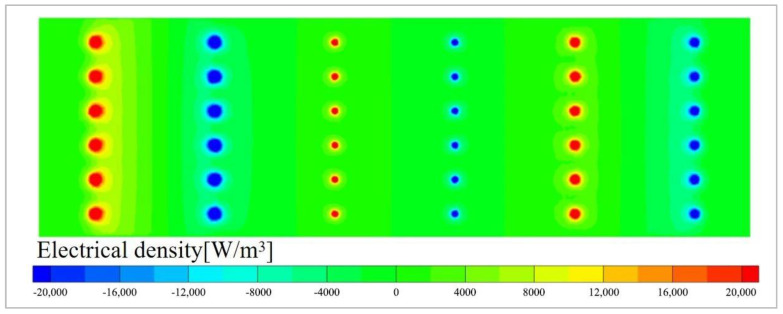
Electrical density distribution of glass furnace in Case-1.

In the electric melting furnace, due to the low electrical conductivity of the glass melt, the electric power is primarily concentrated near the electrodes. Figure 5 shows the electric power density distribution for each scenario. It can be observed that the electric power density is highest at the location of the electrode rods. The further away from the electrodes, the lower the electric power density becomes. In regions far from the electrodes, the electric power density is minimal, and the effect of electrical assistance to melting can be neglected.

The average temperature of the glass melt, calculated from the simulation results of each model, is summarized in Table 4. It can be observed that Case-2 has the highest average glass melt temperature, approximately 57.2 K higher than that of Case-0. However, excessively high temperatures could cause structural damage to the furnace and shorten its service lifespan. Therefore, to ensure the quality of the glass melt, it is advisable to adopt a lower-temperature solution whenever possible to reduce energy consumption.

**Figure 6:**
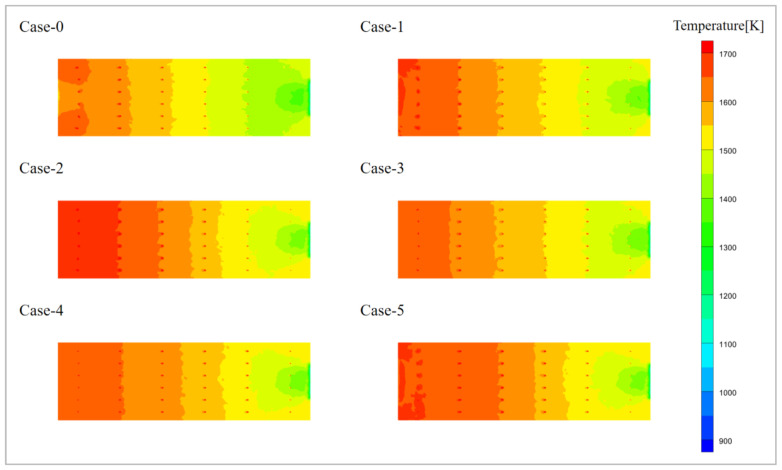
Temperature distribution of liquid glass in Y = 0.35 plane.

Figure 6 shows the temperature distribution of the glass melt on the Y = 0.35 surface. The temperature of the glass melt follows a stratified distribution with respect to the electrode position, decreasing from the kiln head to the kiln tail. This confirms that combustion reactions are still the primary source of heat, and the voltage variations do not cause significant changes in the temperature trend of the glass melt. From the figure, it can be observed that the high-temperature areas for Case-2 and Case-5 are the largest, indicating that setting a high pressure at the kiln tail can significantly increase the temperature of the glass melt.

**Figure 7:**
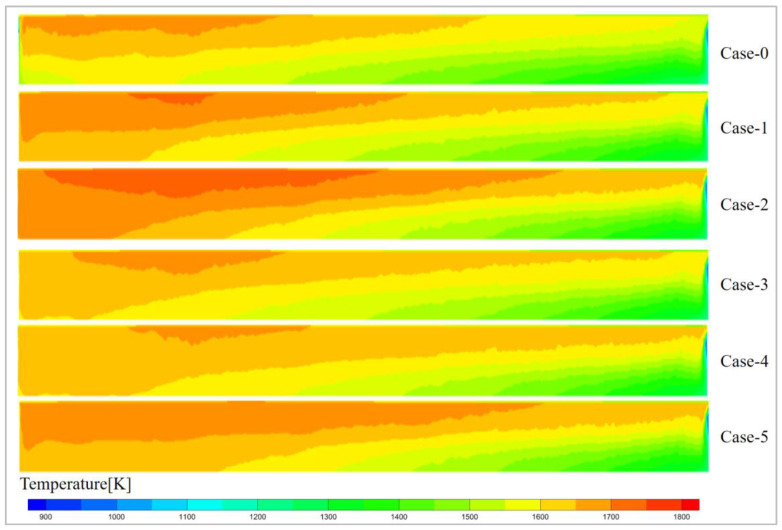
Temperature distribution of symmetrical surface in glass furnace.

Figure 7 shows the temperature distribution on the symmetrical plane of the glass pool kiln. It clearly illustrates the state of heat conduction from the glass surface to the interior of the glass melt, further confirming that the heat in the glass melt is primarily provided by the combustion reaction. The combustion conditions for all six scenarios are identical, with only the voltage settings being changed. Therefore, the temperature changes in the glass melt are primarily caused by voltage variations. From the figure, it can be seen that in the optimized scheme, Case-2 has the largest high-temperature region, while Case-3 and Case-4 have smaller high-temperature regions. The low-temperature region in the original scheme, Case-0, is relatively large, indicating that the voltage arrangement from the kiln head to the kiln tail, with decreasing voltage, overlooks the need for a substantial amount of heat at the kiln tail. This results in a relatively lower glass melt temperature at the kiln tail, which is unfavorable for the subsequent drawing process.

**Figure 8:**
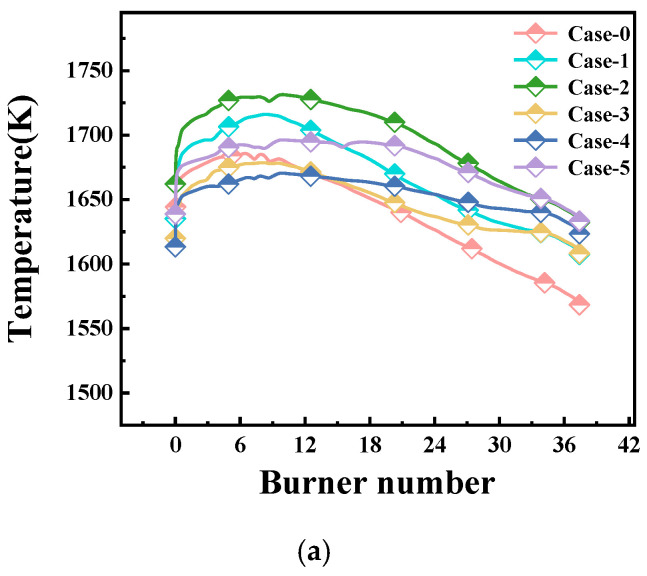

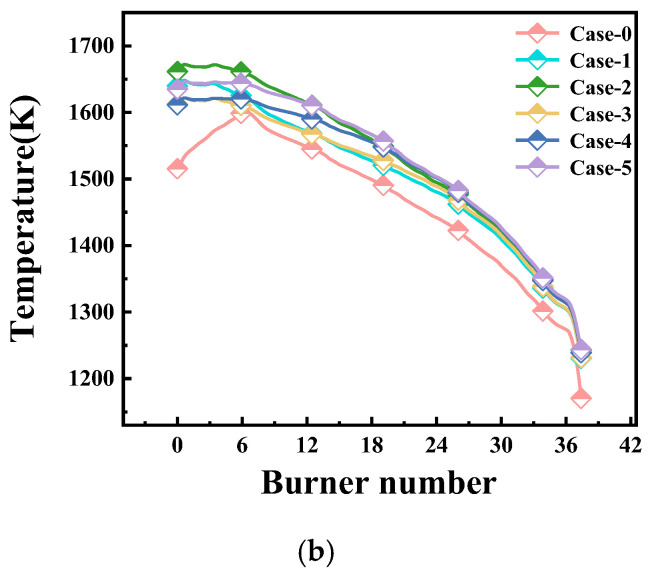
Furnace centerline temperature. (**a**) Surface temperature of molten glass. (**b**) Bottom temperature of molten glass.

The temperature distribution of the glass surface and the centerline temperature at the bottom of the glass pool kiln for the electric boosting melting models (Case-0, Case-1, Case-2, Case-3, Case-4, Case-5) is shown in Figure 8. By analyzing the temperature curve of the glass surface in the pool kiln, it can be seen that the surface temperature of the glass changes proportionally with the electrode voltage input. Compared to Case-0, the surface temperature of the glass is higher in the other schemes, especially in the later stages of the melting kiln. On average, Case-2 has the highest glass surface temperature, followed by Case-5. This indicates that optimizing the input voltage of the electric boosting melting system can help to equalize the temperature field. In the design of the schemes, both Case-0 and Case-1 have the highest voltage input at the kiln head, and the glass surface temperature in Case-1 is about 30 K higher than that in Case-0, indicating that the kiln tail requires more heat. The temperature of the glass melt along the centerline at the bottom of the glass pool generally shows a gradual decrease, with only slight variations, mainly because the overall input power of the furnace has not changed.

### 3.3. Glass Fluid Flow Field

**Figure 9:**
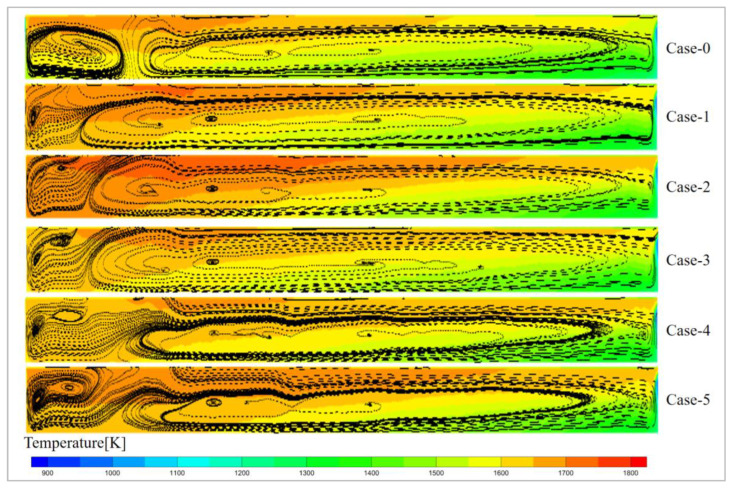
Symmetric surface streamline distribution.

Figure 9 shows the flow streamlines on the symmetrical plane of the glass pool kiln, comparing the flow field distributions of different voltage electric boosting melting models. All six models show two distinct recirculations in the molten glass flow field. The first recirculation occurs at the feed inlet, which is caused by the collision of the feed material with the high-temperature molten glass. This recirculation helps to extend the residence time of the raw materials in the melting zone, enhancing the melting efficiency of the raw materials. The second recirculation is a large-scale recirculation of the molten glass. In this large recirculation, smaller recirculations can be observed at certain locations, which are induced by the heating effect of the electrodes. These small recirculations help to circulate the molten glass vertically, enhancing the clarification and homogenization of the molten glass.

**Figure 10:**
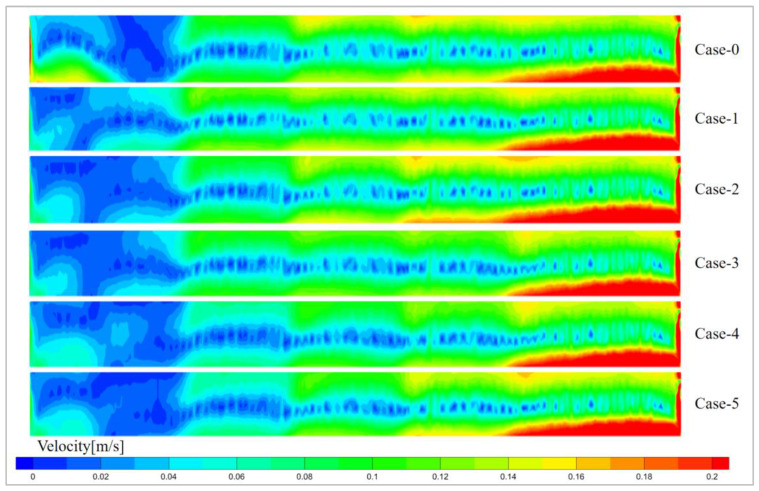
Velocity distribution of symmetry plane.

Figure 10 shows the velocity distribution on the symmetrical plane of the glass pool kiln. The velocity of the molten glass is higher at the glass surface and at the bottom of the glass pool kiln. This is due to the heat sources being the combustion space above the molten glass and the electrode heating at the bottom of the pool kiln, with heat being transferred inward from both the upper and lower directions. Case-2, with the highest overall temperature, also has the highest velocity. This is because higher temperatures result in lower molten glass viscosity, which increases the flow speed. In the electric boosting melting models, Case-0 and Case-1 exhibit the fastest molten glass velocities, followed by Case-2 and Case-3, while Case-5 and Case-4 have the lowest velocities. This is because, in Case-5 and Case-4, the electrodes with the lowest input voltages are located in the feed circulation zone, leading to lower melting efficiency of the raw materials and thus slower molten glass velocities. In contrast, in Case-0 and Case-1, the electrodes with the highest input voltages are placed in the feed circulation zone, enhancing the melting efficiency of the molten glass and increasing its velocity. However, in these cases, the electrodes are more susceptible to erosion by the molten glass, which could shorten their service lifespan.

### 3.4. Residence Time Distribution

Residence time refers to the time it takes for glass particles to move from the molten glass feed inlet to the outlet. In a glass fiber melting furnace, sufficient residence time is required for the complete melting of the glass particles, but excessively long residence times lead to significant energy dissipation. Therefore, residence time is an important indicator for the performance of a glass fiber melting furnace.

**Figure 11:**
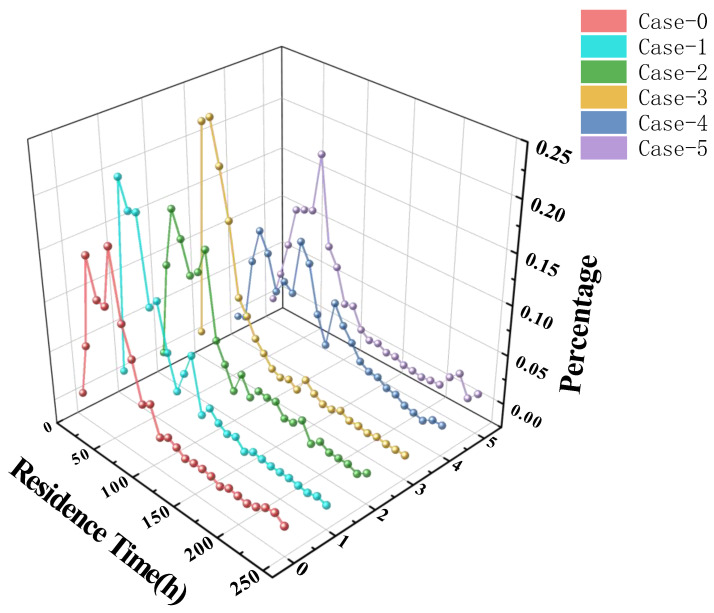
Residence time distribution of glass.

In this study, the residence time distribution of glass particles is shown in Figure 11 and Table 5. The horizontal axis in the figure represents the value for the first ten hours. From Figure 11, it can be seen that the fastest glass particles reach the outlet within 5–10 h after being released from the feed inlet. Within 100 h, most of the particles have already reached the outlet, and almost all particles reach the outlet after 250 h. From Table 5, it is observed that Case-4 has a maximum average residence time of 89.7 h, while Case-3 has the smallest average residence time of 40.5 h. The minimum residence time for Case-0 is 8.0 h, while Case-4 has a minimum residence time of 18.0 h. This is because the electrode voltage distribution in Case-4 increases from the kiln head to the kiln tail, leading to insufficient heat input in the feed circulation area, which requires the most heat.

Under the same conditions, a smaller average residence time is desired, but to ensure the melting quality of the molten glass, a larger minimum residence time is necessary. This suggests that in Case-0, the shorter minimum residence time may lead to a reduction in the glass melting quality.

### 3.5. Quality Factors

Melting factor Equation [19,21]:

(6) $$I_{Mel}=\int_{L}\frac{T}{\mu }dt$$

where *T* and μ represent the temperature and dynamic viscosity of the molten glass at the particle’s position, respectively; *L* is the trajectory of the particle within the furnace; and *t* denotes the time.

Mixing factor Equation:

(7) $$I_{Mix}=\int_{L}\frac{4{\left\|\nabla U\right\|}^{2/3}{D_{r}}^{1/3}}{3\left(0.01^{2/3}\right)}dt$$

where *U* is the velocity of the molten glass at the particle’s position; *D_r_* is the diffusion coefficient (which can be taken as 1.5 × 10^−12^ m^2^/s for ordinary float glass).

The average and minimum values of the melting factor and homogenization factor for the six schemes are shown in Table 6. Although Case-4 has the highest molten glass quality factor, it also has the longest residence time. Considering both the residence time for molten glass melting and the quality factor, the electro-assisted melting models for Case-0, Case-1, and Case-3 exhibit shorter residence times. However, Case-3 has a relatively poor quality factor. Since the molten glass temperature in Case-1 is higher, it can enhance the melting efficiency of the molten glass. At the same time, its average residence time is shorter, and the minimum residence time is slightly longer than that of Case-0. Therefore, it is concluded that the electrode voltage scheme in Case-1 is more reasonable. Specifically, the highest input voltage should be set at the kiln head, while ensuring that the voltage at the kiln tail is also relatively high, as this yields the best molten glass melting quality and the longest furnace lifespan.

## 4. Conclusions

A numerical model coupling the combustion space and glass pool kiln of a 200 t/d typical glass fiber melting furnace was established. The effects of electro-assisted melting input voltage on the temperature field, flow field, and molten glass melting quality in the glass pool kiln were studied and compared. The conclusions are as follows:

(1) When the highest input voltage electrode rods are located in the feeding circulation area, the melting efficiency of the molten glass is enhanced, and the molten glass velocity is higher. At this point, the electrode rods are subjected to the most significant erosion by the molten glass, which may shorten the electrode rod lifespan and cause other related issues.

(2) When the electrode voltage is configured to increase from the kiln head to the tail, the heat input in the feeding zone is insufficient for the initial melting of raw materials. This results in a sluggish flow field and unnecessarily long residence times, which is detrimental to production efficiency.

(3) The optimal scheme involves setting the highest voltage at the kiln head to ensure rapid melting, while maintaining relatively high voltage at the kiln tail to aid the refining process. This configuration balances the thermal history of the melt and minimizes localized erosion, resulting in superior glass quality and an extended furnace service lifespan.

Based on these findings, future research will extend to the optimization of electrode insertion depth. Since the vertical position of electrodes significantly impacts flow patterns and heat release, coupling voltage control with physical electrode configuration will be explored to further enhance energy efficiency and glass homogeneity in industrial applications.

## Figures and Tables

**Table 1:** Fuel ratio of each burner [21].

Burner	1#	2#	3#	4#	5#	6#	7#
Fuel ratio/mass%	11	15	18	21	15	11	9

**Table 2:** Voltage setting.

	Case-0	Case-1	Case-2	Case-3	Case-4	Case-5
1, 2 row electrode voltage/V	250	250	200	200	140	140
3, 4 row electrode voltage/V	200	140	140	250	200	250
5, 6 row electrode voltage/V	140	200	250	140	250	200

**Table 3:** Thermophysical properties of molten glass [22].

Density, ρ/kg·m^−3^	2672.8−0.195·T
Kinematic viscosity, ν/m^2^·s^−1^	exp(−14.31 + 10,711.6/(T − 504.76))
Specific heat, *C_p_*/J·kg^−1^·K^−1^	1052.5 + 0.33 T − 7.42 × 10^−5^·T^2^
Effective thermal conductivity, λ/W·m^−1^·K^−1^	−1.96 + 0.008·T − 6.93 × 10^−6^·T^2^ + 1.736 × 10^−8^·T^3^
Electricity conductivity, σ/Ω^−1^·m^−1^	exp(8.04 − 7781.8/T)

**Table 4:** Average temperature of molten glass.

	Case-0	Case-1	Case-2	Case-3	Case-4	Case-5
Average temperature/K	1570.3	1600.2	1627.5	1590.2	1600.8	1617.8

**Table 5:** Average and minimum residence time.

	Average Residence Time/t·h^−1^	Minimum Residence Time/t·h^−1^
Case-0	62.5	8.0
Case-1	48.5	9.0
Case-2	74.7	9.0
Case-3	40.5	9.0
Case-4	89.7	18.0
Case-5	75.9	13.0

**Table 6:** Melting factor and mixing factor of molten glass.

	Case-0	Case-1	Case-2	Case-3	Case-4	Case-5
Average melting factor	2.17 × 10^8^	2.36 × 10^8^	3.08 × 10^8^	1.91 × 10^8^	4.34 × 10^8^	3.64 × 10^8^
Minimum melting factor	1.67 × 10^7^	3.32 × 10^7^	3.35 × 10^7^	3.26 × 10^7^	7.57 × 10^7^	5.22 × 10^7^
Average mixing factor	1.88	0.76	1.05	0.58	1.57	1.26
Minimum mixing factor	0.18	0.01	0.01	0.01	0.07	0.02

## Data Availability

The original contributions presented in this study are included in the article. Further inquiries can be directed to the corresponding author.
